# Differences in Sleep Disorders between HIV-Infected Persons and Matched Controls with Sleep Problems: A Matched-Cohort Study Based on Laboratory and Survey Data

**DOI:** 10.3390/jcm10215206

**Published:** 2021-11-08

**Authors:** Yen-Chin Chen, Chang-Chun Chen, Patrick J. Strollo, Chung-Yi Li, Wen-Chien Ko, Cheng-Yu Lin, Nai-Ying Ko

**Affiliations:** 1 Department of Nursing, National Cheng Kung University Hospital, College of Medicine, National Cheng Kung University, Tainan 704, Taiwan; yenchin2427@gmail.com; 2 Department of Nursing, College of Medicine, National Cheng Kung University, Tainan 704, Taiwan; windmaxwell@gmail.com; 3 Division of Pulmonary Allergy and Critical Care Medicine, University of Pittsburgh, Pittsburgh, PA 15213, USA; strollopj@upmc.edu; 4 Department of Public Health, College of Medicine, National Cheng Kung University, Tainan 704, Taiwan; cyli99@mail.ncku.edu.tw; 5 Department of Public Health, College of Public Health, China Medical University, Taichung 404, Taiwan; 6 Department of Medicine, College of Medicine, National Cheng Kung University, Tainan 704, Taiwan; winston3415@gmail.com; 7 Department of Otolaryngology, National Cheng Kung University Hospital, College of Medicine, National Cheng Kung University, Tainan 704, Taiwan; 8 Sleep Medicine Center, National Cheng Kung University Hospital, College of Medicine, National Cheng Kung University, Tainan 704, Taiwan

**Keywords:** HIV, sleep disorders, matched controls, sleep-disordered breathing, psychological disturbances, rapid eye movement behavior disorder

## Abstract

Objectives: Sleep disturbances are prevalent problems among human immunodeficiency virus (HIV)-infected persons. The recognition of comorbid sleep disorders in patients with HIV is currently hampered by limited knowledge of sleep-related symptoms, sleep architecture, and types of sleep disorders in this population. We aimed to compare the differences in sleep-related symptoms and polysomnography-based sleep disorders between HIV-infected persons and controls. Methods: The study evaluated 170 men with a Pittsburgh sleep quality index scores greater than 5, including 44 HIV-infected men and 126 male controls who were frequency-matched by sex, age (±3.0 years) and BMI (±3.0 kg/m^2^). For all participants, an overnight sleep study using a Somte V1 monitor was conducted. Differences in sleep-related symptoms and sleep disorders between HIV-infected patients and controls were examined using t-tests or chi-square tests. Results: HIV-infected persons with sleep disturbances more often had psychological disturbances (72.7% vs. 40.5%, *p* < 0.001) and suspected rapid eye movement behavior disorder (25.0% vs. 4.8%, *p* < 0.01) than controls. Sleep-disordered breathing was less common in HIV-infected persons than in controls (56.8% vs. 87.3%, *p* < 0.001). The mean percentage of rapid eye movement sleep was higher among HIV-infected patients than among controls (20.6% vs. 16.6%, *p* < 0.001). Nocturia was more common in HIV-infected persons than in controls (40.9% vs. 22.2%, *p* = 0.02). Conclusions: Psychological disturbances and sleep-disordered breathing can be possible explanations of sleep disturbances in HIV-infected persons in whom sleep-disordered breathing is notable. Further studies are warranted to examine the underlying factors of rapid eye movement behavior disorder among HIV-infected persons with sleep disturbances.

## 1. Introduction

Sleep disturbances are a highly prevalent problem among people living with human immunodeficiency virus (HIV). Almost half of HIV-infected persons worldwide experience sleep disturbances [1]. In Taiwan, HIV-infected persons have been shown to not only exhibit a higher risk of developing sleep disturbances than the general population but also have a 20% higher risk of sleep disturbances than cancer patients [2]. Sleep disturbances encompass a wide range of sleep disorders, including insomnia, sleep-disordered breathing, central disorders of hypersomnolence (e.g., narcolepsy), circadian rhythm sleep-wake disorders, sleep-related movement disorders and parasomnias (e.g., rapid eye movement behavior disorder) [3]. Poor recognition of sleep problems is associated with negative clinical consequences, including a suppressed immune system [4], increased risk of depression [5], poor medication adherence [6,7] and decreased quality of life [8]. Therefore, it is important to understand that antiretroviral therapy patients living with HIV suffer from specific types of sleep disorders. Based on previous studies, we found that sleep disorders among HIV-infected persons do not appear to have been comprehensively explored, but these studies have focused only on insomnia [9] or sleep apnea [10,11,12,13,14].

Polysomnography is a valid diagnostic tool for the assessment of a full range of sleep disorders because it simultaneously records several physiological signals during sleep. As this technique is not limited to only assessing sleep apnea, one can evaluate several sleep conditions when patients undergo an overnight polysomnography test. Currently, we have found sleep-related breathing disorder is prevalent in male HIV-infected persons with sleep complaint [15]. However, there was limited knowledge which specific types of sleep disorders may differentially affect HIV-infected persons compared to controls. To our knowledge, only one small-scale prospective study in HIV-infected persons has evaluated the full range of sleep disorders; the study reported that HIV-infected persons had a significantly higher risk of insomnia than controls (56% vs. 0%), although the risk of having other sleep disorders, such as periodic limb movements and sleep-disordered breathing, was not significantly different from that of controls [16]. Since most sleep disorders can be clinically managed, early detection of sleep-related symptoms and treatment of these problems could prevent negative outcomes. This study aimed to use polysomnography and sleep-related questionnaires to compare the differences in types of sleep disorders as well as sleep-related complaints between persons living with HIV and controls.

## 2. Methods

### 2.1. Study Design

We conducted a case-control study among HIV-infected persons and age-, sex-, and BMI-matched controls who had reported sleep problems (in a 1:3 ratio). This study protocol was approved by the institutional review board of National Cheng Kung University Hospital (NCKUH No. B-BR-104-033). The HIV group was the group of interest, and controls were included for comparison. Informed consent was provided by all HIV-infected participants prior to examination and data collection. If patients were willing to be tested, those interested in enrolling in the study were scheduled to undergo full-channel polysomnography. At the time of fitting the sleep study equipment, subjects completed questionnaires designed to detect sleep disorder-related signs and symptoms.

### 2.2. Study Participants

#### 2.2.1. HIV-Infected Persons with Sleep Disturbances

**Figure 1:**
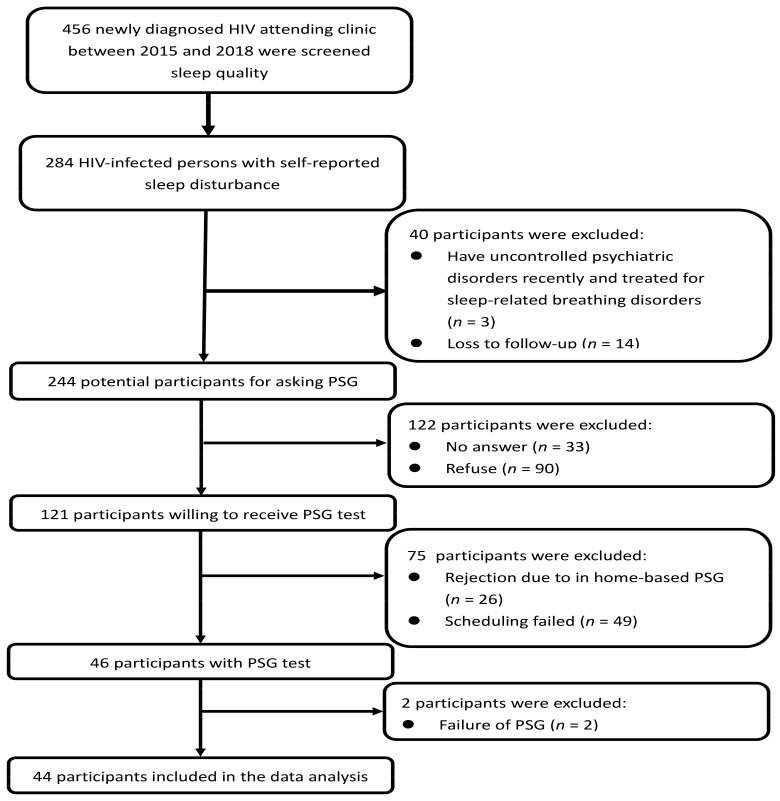
Flowchart of enrollment of HIV-infected persons for the polysomnography test. PSG-polysomnography.

A cross-sectional study was conducted in an HIV clinic in southern Taiwan from 2016 to 2018. The inclusion criteria were subjects who (a) were at least 20 years old, (b) were HIV-infected, and (c) had self-reported sleep disturbances (Chinese version of the Pittsburgh sleep quality index (CPSQI), scores greater than 5). Patients were excluded if they recently were diagnosed with uncontrolled psychiatric disorders (e.g., chronic major depression disorder, dysthymia, generalized anxiety disorder, panic disorder, social phobia, obsessive-compulsive disorder, adjustment disorder, bipolar disorder, schizophrenia, schizoaffective disorder, posttraumatic stress disorder and mental retardation) within the last six months or had the history of treated for sleep-related breathing disorders. A flow chart summarizing the enrollment process for HIV-infected persons is given in Figure 1.

#### 2.2.2. Controls with Sleep Disturbances

Controls were selected from the sleep medicine center database, who came to the hospital seeking treatment for their sleep problems between 2013 and 2017 and had CPSQI scores greater than 5. We excluded persons who had previously been treated with sleep-disordered breathing or whose demographic data were incomplete. Controls were then frequency matched to HIV-infected persons based on sex, age (±3.0 years) and body mass index (BMI) (±3 kg/m^2^).

### 2.3. Measures

#### 2.3.1. Polysomnography Study

An overnight sleep study was performed with a home-based portable Somte polysomnography V1 monitor (Compumedics Sleep, Abbotsville, Australia). Measured signals included an electroencephalogram (C3-A2, C4-A1, O1-O2), bilateral electrooculogram, chin electromyography, electrocardiogram, rib cage and abdominal excursion, nasal airflow via the nasal cannula, oxygen saturation via pulse oximetry and leg movement. Polysomnography data interpretation was performed by accredited sleep technologists in a single reading laboratory following the AASM criteria. Polysomnography reports were verified by a board-certified sleep specialist (C.-Y. Lin).

##### Sleep Architecture

The sleep architecture was calculated by PST-derived measures, which included total sleep time (TST) and sleep latency in minutes, the percent of TST spent in stage 1, sleep 2, stage 3, stage of rapid eye movement sleep, and sleep efficiency. In addition, arousal index was measured.

1. Sleep-disordered breathing:

Sleep-disordered breathing was diagnosed based on having a polysomnography-determined apnea and hypopnea index (AHI) ≥ 5 events/h 3. The AHI is the sum of the number of apneas and hypopneas that occur per hour of sleep, and it has been used as a marker of the severity of sleep-disordered breathing [17].

The following parameters were classified: (1) the apnea index was defined as a ≥90% decrease in airflow over a 10-s period with concomitant respiratory-related chest wall movement for obstructive apnea. (2) The hypopnea index was defined as a ≥30% reduction in baseline airflow for at least 10 s combined with either arousal in an electroencephalogram for ≥3 s or oxygen desaturation ≥ 3%. (3) The OSA index was defined the number of apneas and hypopneas during the study per hour of sleep. (4) The central sleep apnea index is made when apneas without evidence of respiratory efforts constitute 50% or more during the study per hour of sleep. (5) The mixed sleep apnea index was defined as the number of respiratory efforts that were simultaneously categorized as obstructive and central during the study per hour of sleep.

2. Periodic limb movements

Limb movement was scored in accordance with the American Academy of Sleep Medicine (AASM) criteria [18], with a duration of limb movement between 0.5 and 10 s and a > 8 µV amplitude increase from baseline in a leg electromyogram channel. Limb movement was not scored if it occurred within (before or after) 0.5 s of the end of apnea, hypopnea, or respiratory effort-related arousal. An arousal and a limb movement were considered to be associated with each other if there was <0.5 s between the end of one event and the onset of the other, regardless of which came first [18]. Periodic limb movements were defined as a series of at least four limb movements with an interval between limb movements being more than 5 s but less than 90 s [19]. The periodic limb movement index was calculated as the total number of periodic limb movements per hour of sleep. The diagnosis of periodic limb movement in sleep was defined as having >5 events per hour.

3. Suspected rapid eye movement behavior disorder

Rapid eye movement behavior disorder diagnosis was based on the criteria of the third edition of the International Classification of Sleep Disorders (ICSD-3) [3]. Rapid eye movement behavior disorder includes (1) the presumption that it arises from rapid eye movement sleep based on reports of dream enactment and (2) evidence of rapid eye movement sleep without atonia on polysomnography, defined as excessive muscle activity during more than 10% of rapid eye movement sleep epochs.

To define excessive muscle activity, we used the criteria from the AASM scoring manual [18]. Sustained muscle activity (tonic activity) was assessed in 30-s epochs. If electromyography activity in an epoch of rapid eye movement sleep exceeded twice that of the background activity level for more than 50% of the epoch, it was considered to be excessive muscle activity. Excessive transient muscle activity (phasic activity) was measured in 3-s mini-epochs during rapid eye movement sleep and defined as submental electromyography activity bursts lasting 0.1–5 s and exceeding four times that of the background level.

#### 2.3.2. Questionnaire

##### Sleep-Related Physical Symptoms

Sleep-related physical symptoms were measured with a seven-item scale with dichotomous responses (yes/no) that included snoring, nonrestorative sleep, dry mouth when waking up, excessive daytime sleepiness, morning headache, nocturia and sleepwalking. Nocturia was defined as “the complaint that the individual wakes up one or more times per night to void” [20].

##### Poor Sleep Quality

Poor sleep quality was identified by the CPSQI. The CPSQI is a 19-item questionnaire. If the participants had a total CPSQI score greater than 5, they were considered to have poor sleep quality [21].

##### Psychological Disturbances

Psychological disturbances (anxiety and/or depression) were characterized by abnormal results on the hospital anxiety and depression scale [22,23]. The hospital anxiety and depression scale contain 14 items, each scaled from 0 to 3 points, meaning that a person could score between 0 and 21 points for either anxiety or depression. Scores over 8 were considered to reflect anxiety or depression [22]. The scale showed a positive predictive value of 96.7% among HIV-infected persons [24].

#### 2.3.3. Clinical Data Collection

Baseline clinical variables associated with sleep outcomes, including age, BMI, neck circumference, level of education, occupation, use of hypnosis, and comorbidities (rhinitis/sinusitis, gastroesophageal reflux disease, hypertension, hyperuricemia, asthma, angina, hyperlipidemia and diabetes mellitus), were collected. HIV-related clinical data were retrieved from patients’ electronic medical records, including time since HIV diagnosis, delayed diagnosis (if CD4 counts at HIV diagnosis ≤ 200 cells/mm^3^), viral load of PSG testing (if viral load was fewer than 20 copies/mL, it was defined as undetectable), use of antiretroviral therapy, years from antiretroviral therapy initiation, and substance use (tobacco, illegal drugs, and alcohol).

### 2.4. Data Analysis

Participant characteristics and sleep-related complaints from polysomnography data were calculated based on HIV status. The HIV-infected persons and controls were descriptively compared using chi-square tests for categorical measures and t-tests for continuous measures. Logistic regression analysis was used to determine whether sleep-related physical and psychological symptoms were associated with sleep disorders. The 95% confidence intervals that did not contain the null hypothesis value were defined as statistically significant. Analyses were conducted in the SAS 9.4 (SAS, Cary, NC, USA) statistical package.

## 3. Results

### 3.1. Demographics

A total of 44 HIV-infected men were recruited from an HIV clinic in southern Taiwan from 2016 to 2018. The HIV group was all male, with an average age of approximately 35 years old, and the majority of their BMI scores were lower than 24 kg/cm^2^. Data from 126 matched controls were obtained from the sleep medicine center database. A total of 170 men were analyzed, including 44 men in the HIV group and 126 men in the control group. There were no significant differences in demographics, use of hypnosis or comorbidities between groups, except for rhinitis/sinusitis. The mean age was 34.2± 9.1 years in the HIV group and 35.6 ± 10.0 years in the control group. The mean BMI was 23.6 ± 4.0 kg/cm^2^ in the HIV group and 23.1 ± 4.0 kg/cm^2^ in the control group. A total of 90.9% of HIV-infected persons had received antiretroviral therapy, with a median CD4 count (SD) of 557.5 (230.8) cells/mm^3^ and 27.3% detectable viral load with a mean viral load (SD) of 17,749.6 (103,463.3) copies/mL (Table 1).

### 3.2. Differences in Sleep Architecture

Compared to the controls, the HIV-infected persons had a significantly higher average total sleep time, a lower percentage of stage 1 sleep, a greater percentage of REM stage sleep, and a lower average arousal index (Table 2). When we used the normal sleep architecture as the cutoff point for evaluating the difference in sleep architecture and arousal index between the two groups, we found that there were significant increases in the percentage of HIV-infected persons compared to controls with stage 2 ≥ 50% (72.7% vs. 54.8%) and REM sleep ≥ 25% (20.5% vs. 9.5%). The arousal index was significantly higher in the control group than in the HIV patient group (30.4 versus 18.2, *p* < 0.001) (Table 2).

### 3.3. Types of Sleep Disorders

Psychological disturbances (72.7% vs. 40.5%, *p* < 0.001) and suspected rapid eye movement behavior disorder (25.0% vs. 4.8%, *p* < 0.001) were more frequent in the HIV group than in the control group. The rates of sleep-disordered breathing were 56.8% and 87.3% in the HIV and control groups, respectively (*p* < 0.001) (Table 3). Specifically, apnea, hypopnea, and the mixed obstructive index were significantly higher in the controls than in those in the HIV group.

### 3.4. Differences in Sleep-Related Complaints

**Figure 2:**
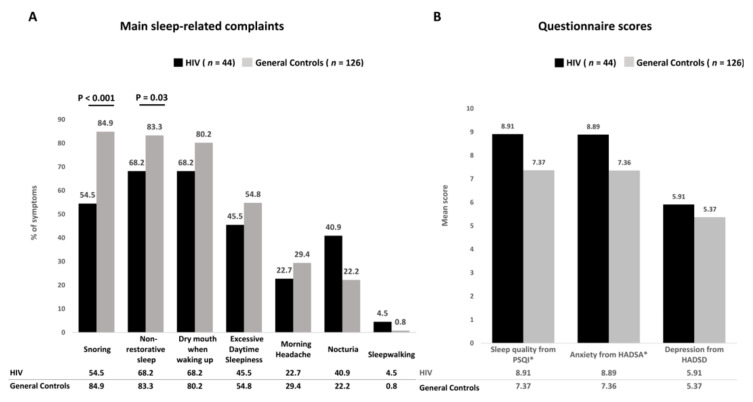
Frequency of main sleep-related complaints (**A**) and questionnaire scores (**B**) among HIV-infected persons compared with controls. Abbreviations: PSQI—Pittsburgh sleep quality index; HADSA—Hospital Anxiety and Depression Scale for anxiety; HADSD—Hospital Anxiety and Depression Scale for depression.

Snoring, nonrestorative sleep, and dry mouth upon waking up were the three main sleep-related complaints among the 170 study subjects. Nocturia was more frequent in the HIV group than in the control group (40.9% vs. 22.2%, *p* = 0.02) (Figure 2A). The controls reported higher prevalence rates of snoring (84.9% vs. 54.5%, *p* < 0.001) and nonrestorative sleep (83.3% vs. 68.2%, *p* = 0.03) than HIV-infected persons (Figure 2B).

## 4. Discussion

We conducted the first comparative study in Asia that evaluated a wide spectrum of sleep disorders based on polysomnography results in patients with sleep disturbance with and without HIV. We observed that HIV-infected persons experienced more psychological disturbances than the controls.

Our results were similar to those of a prior study that reported that HIV-infected persons have greater average State-Trait Anxiety Inventory scores than controls [16]. Given the bidirectional correlation between poor sleep quality and depression or anxiety [26], provision and management of psychological care may help improve sleep quality. We suggest that early detection and treatment of psychological disturbances might improve sleep quality and that addressing sleep disturbances may relieve psychological morbidity.

Regarding sleep patterns, HIV-infected persons exhibited an increased percentage of REM sleep compared to controls. This result was similar to a previous finding by Gallego and colleagues (2004), who found that the percentage of REM sleep in HIV-infected persons using efavirenz without insomnia was 22.6% [27]. Psychological disturbances might be a major risk factor explaining increases in the percentage of REM sleep because psychological disturbances can induce alterations in sleep stages such as increased sleep onset latency and increased percentage of REM sleep [28]. On the other hand, the use of antiretroviral therapy, in particular, nevirapine regimen [29] and efavirenz [30], might be a factor explaining increases in the percentage of REM sleep due to induced central nervous system arousal, including vivid dreams. Future research could evaluate which precipitating factors are associated with changes in sleep architecture among HIV-infected persons.

Our study is the first to show a higher proportion of suspected rapid eye movement behavior disorder in HIV-infected persons than in controls. The underlying possible rapid eye movement behavior disorder pathogenesis may include HIV infection [31] and medication use [32], both of which may lead to neural damage that results in neurodegenerative disorders. Rapid eye movement behavior disorder has been recognized to be highly correlated with neurodegenerative disorders such as Parkinson’s disease [33]. Parkinsonism is the most common movement disorder in HIV-infected persons, and it may be a manifestation of early neurodegeneration [34,35]. Further research is needed to reveal the mechanisms responsible for the relationship between HIV infection and rapid eye movement behavior disorder.

The study findings showed a lower rate of sleep-disordered breathing (AHI ≥ 5) in HIV-infected persons than in the controls. This finding is consistent with a prior analysis, which found that HIV-positive men with antiretroviral therapy treatment had less sleep-disordered breathing than HIV men without antiretroviral therapy and HIV-negative men (70.7% vs. 73.2 and 86.7%, respectively) [11]. The fact that the control group had a higher prevalence of sleep-disordered breathing than the HIV-infected persons may be partially explained by the fact that the controls had more reported rhinitis/sinusitis and sleep complaint symptoms such as snoring than those in the HIV group. Previous studies have found that snoring and sleep apnea are highly correlated [36], and persons with rhinitis/sinusitis have been found to have a 7.6-fold greater chance of sleep apnea syndrome than those without rhinitis/sinusitis [37]. Since both rhinitis/sinusitis and snoring are obstructive diseases of the upper and lower airways, this could have caused our controls to have a higher probability of risk for sleep-disordered breathing than those in the HIV group.

There was a notable observation that the prevalence of nocturia in HIV-infected persons was two-fold higher than that in the controls. Limited data on nocturia in HIV-infected persons are available. The pathophysiology of nocturia remains unclear, but the data available to date suggest that nocturia is possibly due to three associated mechanisms: nocturnal polyuria, bladder overactivity, and sleep disorders, which could be of urological, neurological, or psychological origin [38]. Future research is needed to further evaluate the relationship between HIV infection and nocturia.

Previous study has demonstrated that in-lab or in-home PSG were similar recording quality and failure rate. In addition, the apnea-hypopnea index also was similar between home and sleep laboratory setting [39]. The overall attrition rate of our study was 62% in HIV-infected persons who were willing to received PSG test. Only 36.4% (44/121) of subjects completed the home-based PSG test. In 62% (75/121) of the attrition due to unable schedule an available time to do the PSG (40.5%) and the unattended home-based PSG was not the patients’ preference (21.5%). Those not preferring an in-home PSG set-up cited signal loss as the prime concern. In our review of the current literature, there are no previous studies addressing this attrition rate of unattended home-based PSG in HIV populations. Our findings are similar to those of Angela, who reported that 25.0% of patients preferred to have the sleep study at the laboratory due to fewer distractions and trained staff staying with them [40]. Our data show that using home set-up PSG for early diagnosis OSA in HIV-infected persons still has obstacles due to lower acceptance rate.

Our study has some limitations. First, our HIV participants consisted of young males with normal BMI, which may limit the generalizability to the overall HIV-positive population. Second, our controls selected from the sleep medicine center could not represent the general population since all of them were treatment seekers. Second, comparisons of both sleep architecture and specific sleep disorders between HIV-infected persons and controls might have underscored the sleep disturbance of HIV patients, since people in the control group also suffered from sleep problems. The prevalence of sleep problems in HIV patients is, therefore, expected to be greater than that in the general population. Finally, psychological disturbances were measured using the hospital anxiety and depression scale, which was unable to provide objective evidence to confirm psychological disturbances. It is suggested that future studies should consider using objective measures, such as heart rate variability or autonomic nervous system testing, for further validation of psychological distress in patients.

## 5. Conclusions

We observed that psychological disturbances and sleep-disordered breathing were more prevalent in HIV-infected persons with sleep disturbances. HIV-infected persons showed a higher rate of psychological disturbances and suspected rapid eye movement behavior disorder than matched controls. There were significant increases in the mean percentage of REM sleep in HIV-infected persons compared to matched controls. Additionally, nocturia was found to be more likely to occur in HIV-infected persons than in controls. The underlying factors responsible for the higher prevalence of psychological disturbances and rapid eye movement behavior disorder among HIV-infected persons with sleep disturbance warrant further investigation.

## Figures and Tables

**Table 1:** Participant demographics (*n* = 170).

Variables	HIV, *n* = 44	Controls, *n* = 126	*p* Value
Age (year) [mean, SD]	34.2, 9.1	35.6, 10.0	0.40
Body mass index (kg/cm^2^) [mean, SD]	23.6, 4.0	23.1, 4.0	0.44
Neck circumference (cm), [mean, SD]	36.3, 2.2	36.3, 2.5	1.00
Education level			0.11
Under junior	3 (6.8)	5 (4.2)	
Senior/college	38 (86.4)	90 (75.6)	
University and above	3 (6.8)	24 (20.2)	
Occupation			0.17
Non-shift worker	31 (79.5)	79 (88.8)	
Shift worker	8 (20.5)	10 (11.2)	
Use of hypnosis	10 (22.7)	23 (18.3)	0.52
Comorbidities			
Rhinitis/sinusitis	14 (31.8)	67 (53.2)	0.02
Gastroesophageal reflux disease	18 (40.9)	45 (35.7)	0.54
Hypertension	3 (6.8)	15 (11.9)	0.35
Hyperuricemia	2 (4.5)	11 (8.7)	0.37
Asthma	4 (9.1)	10 (7.9)	0.81
Angina	1 (2.3)	9 (7.1)	0.24
Hyperlipidemia	3 (6.8)	7 (5.6)	0.76
Diabetes mellitus	0 (0.0)	1 (0.8)	0.55
Use of ART	40 (90.9)	-	-
Years since ART initiation [mean, SD]	1.9, 2.0	-	-
≤1 year	14 (35.0)	-	
1–3 years	19 (47.5)	-	
>3 years	7 (17.5)	-	
CD4 count (copies/mL) [mean, SD]	557.5, 230.8	-	-
≤200	12 (28.6)	-	
>200	30 (71.4)	-	
Viral load (copies/mL) [median, SD]	17,749.6, 103,463.3	-	-
Undetectable	32 (72.7)		
Detectable	12 (27.3)		
Year since HIV diagnosis [mean, SD]	2.7, 3.0	-	-
≤1 year	15 (34.1)	-	
1–3 years	17 (38.6)	-	
>3 years	12 (27.3)	-	

Note: ART—antiretroviral therapy, HIV—human immunodeficiency virus, SD—standard deviation.

**Table 2:** Differences in sleep architecture by PST-derived measures between HIV-infected persons and controls.

Variables	HIV (*n* = 44)	Controls (*n* = 126)	*p* Value
	*n* (%)		
Total sleep time (min)			
Mean, SD	408.7, 95.9	356.4, 50.3	<0.001
Sleep latency (min)			
<30 ^a^	39 (88.6)	117 (92.9)	0.38
Mean, SD	19.8, 55.4	12.5, 13.4	0.39
Stage 1 (%)			
≥5% ^a^	42 (95.5)	123 (97.6)	0.46
Mean, SD	15.7, 8.4	21.8, 14.5	<0.01
Stage 2 (%)			
≥50% ^a^	32 (72.7)	69 (54.8)	0.04
Mean, SD	54.0, 9.5	50.9, 11.1	0.10
Stage 3 (%)			
≥20% ^a^	5 (11.4)	21 (16.7)	0.40
Mean, SD	9.7, 8.6	10.7, 9.1	0.54
Rapid eye movement stage (%)			
≥25% ^a^	9 (20.5)	12 (9.5)	0.05
Mean, SD	20.6, 5.4	16.6, 6.6	<0.001
Sleep efficiency (%)			
≥85% ^a^	30 (68.2)	77 (61.1)	0.40
Mean, SD	86.2, 10.6	84.7, 10.8	0.43
Arousal index (events/hour)			
≥16.8 ^b^	19 (43.2)	103 (81.7)	<0.001
Mean, SD	18.2, 11.8	30.4, 14.5	<0.001

^a^ The cutoff point for normal sleep architecture was based on the AASM criteria; ^b^ The cutoff point for the arousal index was based on the findings of Bonnet M & Arand D (2007) due to arousal index variability by age [25]; Note: SD—standard deviation.

**Table 3:** Sleep disorders in HIV-infected persons and controls.

Variables	HIV (*n* = 44)	Controls (*n* = 126)	*p* Value
	*n* (%)	*n* (%)	
Sleep-disordered breathing (apnea-hypopnea index ≥ 5)	25 (56.8%)	110 (87.3%)	<0.01
Apnea index (events/hour) [mean, SD]	3.39, 8.72	6.80, 10.47	0.04
Hypopnea index (events/hour) [mean, SD]	8.53, 9.24	17.83, 15.07	<0.001
Obstructive sleep apnea index (events/hour) [mean, SD]	2.91, 8.75	5.57, 8.86	0.09
Central sleep apnea index (events/hour) [mean, SD]	0.38, 0.52	0.28, 0.51	0.25
Mixed apnea index (events/hour) [mean, SD]	0.09, 0.25	0.99, 3.09	<0.01
Psychological disturbances	32 (72.7%)	51 (40.5%)	<0.001
Rapid eye movement behavior disorder	11 (25.0%)	6 (4.8%)	<0.01
Periodic limb movements	5 (11.4%)	18 (14.3%)	0.23

## Data Availability

Not applicable.

## Abbreviations

AASM	American Academy of Sleep Medicine
AHI	apnea and hypopnea index
BMI	body mass index
CPSQI	the Chinese version of the Pittsburgh sleep quality index
HIV	human immunodeficiency virus
ICSD-3	the International Classification of Sleep Disorders—third edition
OSA	obstructive sleep apnea
